# Suicide Safety Planning: Clinician Training, Comfort, and Safety Plan Utilization

**DOI:** 10.3390/ijerph17186444

**Published:** 2020-09-04

**Authors:** Emma H. Moscardini, Ryan M. Hill, Cody G. Dodd, Calvin Do, Julie B. Kaplow, Raymond P. Tucker

**Affiliations:** 1 Department of Psychology, Louisiana State University, 216 Audubon Hall, Baton Rouge, LA 70803, USA; rtucker1@lsu.edu; 2 Baylor College of Medicine and Texas Children’s Hospital, 1102 Bates Ave., Suite C.0235.05, Houston, TX 77030, USA; Ryan.Hill@bcm.edu (R.M.H.); cododd@utmb.edu (C.G.D.); calvindo0710@gmail.com (C.D.); Julie.Kaplow@bcm.edu (J.B.K.)

**Keywords:** suicide, safety planning, suicide prevention

## Abstract

Extant literature has demonstrated that suicide safety planning is an efficacious intervention for reducing patient risk for suicide-related behaviors. However, little is known about factors that may impact the effectiveness of the intervention, such as provider training and comfort, use of specific safety plan elements, circumstances under which providers choose to use safety planning, and personal factors which influence a provider’s decision to use safety planning. Participants were (*N* = 119) safety plan providers who responded to an anonymous web-based survey. Results indicated that most providers had received training in safety planning and were comfortable with the intervention. Providers reported that skills such as identifying warning signs and means safety strategies were routinely used. Providers who reported exposure to suicide were more likely to complete safety plans with patients regardless of risk factors. In addition, almost 70% of providers indicated a need for further training. These data provide important considerations for safety plan implementation and training.

## 1. Introduction

Suicide is the 10th leading cause of death in the United States (U.S.), accounting for approximately 45,000 deaths per year [1]. Suicide attempts are even more frequent, with an estimated 1.3 million suicide attempts among U.S. adults each year [2] at an estimated cost of $93.5 billion due to factors such as injury treatment costs and loss of productivity [3]. To help combat this public health crisis, the National Strategy for Suicide Prevention advocates for increased use of empirically supported interventions to reduce suicide risk in a variety of settings [4]. Of the existing preventative interventions for reducing suicide risk, perhaps the most widely used is suicide safety planning, yet little is known regarding the implementation of suicide safety planning in clinical practice. An accurate understanding of provider impressions of suicide safety planning is needed to inform further dissemination efforts and to improve safety planning education and training. This paper presents the findings of a survey of providers who use safety planning so as to evaluate provider training and comfort with the intervention, factors associated with decisions to use safety planning, and safety planning quality. 

The Suicide Prevention Resource Center and the Joint Commission, among others, recommend safety planning as a standard of care for individuals identified as at-risk for suicide-related behaviors [5,6]. Safety planning is a brief intervention to help individuals survive suicidal crises by having them develop a set of steps to reduce the likelihood of engaging in suicidal behavior. Safety planning is frequently included as an element in cognitive-behavioral interventions for suicide prevention and can also be used as a brief standalone intervention, typically paired with referral for mental health treatment [7,8,9]. Safety planning involves a collaborative process by which a therapist and patient identify a series of action steps to be taken in the event the patient experiences suicide ideation, arranged in order of increasing response intensity. A manualized version of safety planning, the Safety Planning Intervention [10], includes identifying (a) warning signs or triggers that indicate suicide ideation is likely to occur, (b) internal coping strategies (e.g., distracting activities) for use when those triggers occur or when experiencing suicide ideation, (c) social contacts for further distraction or social locations that may provide distraction, (d) supportive contacts (who can provide assistance), (e) emergency resources (e.g., therapist phone numbers, hotlines, local hospital emergency room locations), and (f) steps to ensure the safety of the home environment that minimize the patient’s ability to act on suicidal thoughts or urges (i.e., reducing access to lethal means) [10].

Studies of suicide safety planning support the efficacy of the intervention for reducing patient risk for suicide-related behaviors. In a recent study, patients who received the Safety Planning Intervention and follow-up phone contact in an emergency department setting were nearly half as likely to make a suicide attempt in the six months following hospitalization, as compared with individuals provided usual care [11]. In an examination of the SAFE VET program, which consists of a safety planning followed by structured follow-up calls, 61% of respondents reported using the safety plan to reduce suicide risk [12]. In a study of active duty U.S. Army personnel, use of crisis response planning (an intervention highly similar to safety planning) was associated with a faster decline in suicide ideation and fewer inpatient hospitalization days as compared with a safety contract intervention [13]. Crisis response planning, like safety planning, is a collaboratively derived plan to help ensure patient safety outside of healthcare visits. In this intervention, patients are provided with a notecard to write down the following: personal warning signs, self-management strategies, reasons for living, social supports, and crisis supports. Notably, crisis response planning omits a discussion of means safety, which may impact a provider’s decision to use crisis response planning versus safety planning. Providers may select crisis response planning if time is limited or they feel discomfort discussing means safety with patients. Alternatively, providers may select safety planning to complete both elements in a single session (e.g., in an Emergency Department). Brief Cognitive Behavioral Therapy, for example, recommends that crisis response planning and means safety counseling occur separately but in succession [14].

Although these data are promising, other studies point toward factors that may impact the efficacy of safety planning. For example, higher quality safety plans have been associated with fewer psychiatric hospitalizations among veterans [15]. Higher quality safety plans have been characterized by the aspects of the safety plan completed (e.g., a lower quality safety plan would omit a section such as means safety or internal coping skills) as well as the specificity of the plan (e.g., a lower quality safety plan would list an internal coping strategy as “read” instead of “read A Tale of Two Cities”) [15]. Thus, ensuring that safety plans are of high quality may be crucial to the efficacy of the intervention. Similarly, the method of administration may impact safety planning efficacy: In a study of adults who received a self-administered safety plan (i.e., completed without the assistance of a mental healthcare provider) during a visit to the Emergency Department, only 37.4% reported having received a safety plan during a follow-up call [16]. Thus, self-administration of safety plans may result in a lower rate of safety plan completion or retention. Finally, prior research indicates that clinical decision-making regarding the treatment of suicide-related behaviors can be impacted by a myriad of personal factors of the provider [17,18]. For example, research has demonstrated that the more religious a clinician reports being, the more likely they are to believe a patient requires hospitalization due to suicide risk [17]. In addition, research indicates that Posttraumatic Stress Disorder symptoms in clinicians are related to lower likelihood of believing a patient is in need of hospitalization [18]. As such, personal factors may also influence when providers decide to initiate safety planning if a clear protocol for when this should occur is not detailed. 

Despite this emerging evidence, little is known about how safety planning is commonly used and implemented in clinical settings, and additional research is needed to evaluate how provider factors might impact decisions regarding safety plan utilization. For example, research has demonstrated that most hospital staff perceive safety planning to be helpful and increased their comfort in discharging patients believed to be at risk for suicide [19]. In contrast, although Reyes-Portillo and colleagues found that most providers had positive views regarding safety planning, about half of providers did not believe that safety planning was useful for patients believed to be at high risk for suicide, potentially affecting provider decision-making regarding safety plan use [20]. An accurate understanding of how safety planning is used in clinical settings can inform efforts to develop tools to improve the quality and efficacy of safety planning, establish best-practice parameters, and develop needed training materials. In addition, understanding provider factors that may influence safety planning decision-making is vital. As a first step toward addressing this gap in the empirical literature, this study sought to provide data on the use of safety planning in clinical settings. Specifically, this study sought to gather data on (a) provider training and comfort with safety planning; (b) use of safety planning elements; and (c) the circumstances under which providers decide to use safety planning. With respect to provider use of safety planning, we hypothesized that greater overall provider exposure to suicide, greater comfort with safety planning, more exposure to training in safety planning, and having an institutional policy or protocol regarding suicide safety planning would all be associated with greater use of safety planning in a variety of circumstances.

## 2. Materials and Methods 

### 2.1. Participants 

Participants (*n* = 119) were safety plan providers, 24–71 years of age (*M* = 39.24, *SD* = 12.56 years) who responded to an anonymous web-based survey targeting “Any mental health service provider (18 years of age or older) with experience in suicide safety planning.” Participants were recruited via distribution to email listserv members and word-of-mouth. Inclusion criteria were: 18 years of age or older and self-reported experience with suicide safety planning. 

Participants (74.2% female) had between one and 44 years of experience providing mental health services (*M* = 11.90, *SD* = 10.89). Participants reported their race/ethnicity as non-Hispanic White (74.2%), Hispanic (10.8%), African American or Black (4.3%), Asian American (4.3%), and other (6.5%). The majority of participants were psychologists (34.4%), followed by students (20.4%; of whom *n* = 14 were clinical psychology students), therapists/counselors/psychological associates (19.4%), social workers (9.7%), crisis center workers (5.4%), psychiatrists (2.2%), school/guidance counselors (1.1%), and others (7.5%). 

Participants primarily practiced in outpatient/private practice/community clinics (49.4%), followed by crisis centers (20.7%), academic medical centers (10.9%), inpatient/psychiatric hospitals (4.3%), school counseling departments (2.2%), and other settings (8.2%). Participants had completed a doctoral degree (40.9%), a Master’s degree (47.3%), a Bachelor’s degree (10.8%), and an Associate’s degree (1.1%).

### 2.2. Procedures 

All subjects gave their informed consent for inclusion before they participated in the study. The study was conducted in accordance with the Declaration of Helsinki, and the protocol was approved by the appropriate Institutional Review Board. Providers who received the advertisement email reviewed a letter containing the elements of informed consent and indicated their consent to participate by completing the online survey. The survey required approximately 20 min to complete, and no compensation was provided. 

### 2.3. Measures

The survey was developed specifically to address the hypotheses of this study. As a result, psychometric data are not available. The survey included sections on provider’s practice data, safety planning experience and training, safety plan use as a clinical tool, and patient obstacles to safety planning.

Provider’s practice data included demographic data (participant age, gender, race/ethnicity, and highest degree completed) and information about the scope and setting of their practice (occupation, primary setting, years of experience in providing mental health services, patient population). Moreover, in this section, the survey asked providers to provide information regarding their personal exposure to suicide (e.g., amongst their friends, family, and colleagues, and personal lived experience with suicidal thoughts and behaviors) as well as professional exposure to suicide (i.e., losing a patient to suicide). 

The safety planning experience and training section included questions about having received training in safety planning and whether or not that training was a part of their educational curriculum, familiarity with the manualized Safety Planning Intervention, frequency of safety plan use with patients, comfort providing safety planning, and whether participants felt the need for additional training in safety planning. 

The safety plan use section included the Provider’s estimate of the time required to complete a safety plan and the presence of an institutional standard for safety planning. In addition, in this section, participants were asked to estimate how often they included each of a series of elements in their safety planning process (e.g., “When creating a safety plan, how often do you … include individual coping skills?) The first seven elements captured the core elements of safety planning as outlined by Stanley and Brown [10], whereas the remaining elements captured other aspects of safety planning that might be useful or constitute good clinical practice (e.g., use of a Hope Box, practicing use of the safety plan with patients, including family members in the process, etc.) Finally, participants were asked to select, from a list of examples, which situations they would likely create a safety plan in (e.g., “When a patient has developed a suicide plan” or “When patients have thoughts about ending their lives by suicide.”). Please see Supplementary Materials for the study questionnaire.

## 3. Results

### 3.1. Provider Training and Comfort with Safety Planning

The majority (87.9%) of participants reported receiving training in safety planning, and 51.7% reported receiving this training in safety planning as part of their formal education. Approximately a third of participants (34.5%) reported familiarity with the free, online manual for safety planning [10]. Years of experience providing mental health services were not significantly related to receiving training in safety planning (*r* = 0.16, *p* = 0.13) but were significantly, positively related to receiving safety planning training as part of one’s education (*r* = 0.37, *p* < 0.01). The majority of participants reported being extremely comfortable (43.7%) or somewhat comfortable (45.4%) with safety planning for suicide prevention, whereas a minority reported being somewhat uncomfortable (8.4%) and extremely uncomfortable (2.4%) with safety planning for suicide prevention. The majority of participants (68.1%) indicated a desire for additional training in safety planning. 

### 3.2. Inclusion of Safety Plan Elements

To make inferences about safety plan quality, clinicians were asked to estimate what percent of the time they included various components when creating a safety plan with a patient. Results are displayed in Table 1. The most frequently included components were contact information for emergency resources (*M* = 97.34, *SD* = 8.65), individual coping skills (*M* = 94.58, *SD* = 12.36), and social coping skills or social distraction (*M* = 90.32, *SD* = 16.32). The least frequently included components were creation of a hope box (*M* = 12.23, *SD* = 23.86) or virtual hope box (*M* = 12.72, *SD* = 25.09) and practicing use of the safety plan in session (*M* = 46.07, *SD* = 39.75). Clinicians reported that, on average, safety plans are completed in 24.71 min (*SD =* 20.00 min). 

### 3.3. Circumstances in Which Providers Use Safety Planning

With regard to the decision to implement safety planning, clinicians were provided with example situations (i.e., patient risk levels) and asked to mark all situations in which they would use safety planning. Results are provided in Table 2. The most commonly endorsed situation for utilizing safety plans was when patients have thought about ending their lives by suicide (86.7%), followed by when a patient has identified a method for suicide (73.5%), when a patient has developed a suicide plan (71.7%), and when a patient has begun preparing for suicide (67.3%). Only 50.4% of clinicians reported using safety planning when patients have thoughts about wanting to die, but not wanting to die by suicide. A minority (11.5%) of clinicians used safety planning with all patients. Additionally, 77.9% of clinicians reported completing a safety plan when a patient has previously made a suicide attempt and is having thoughts of suicide, and 41.6% of clinicians reported completing a safety plan when a patient has previously made a suicide attempt but has not had recent thoughts of suicide.

With regard to factors associated with safety plan use: Training in safety planning use and familiarity with the Stanley and Brown [10] manual were not significantly associated with safety plan use in any circumstances. Comfort with safety planning was significantly correlated with frequency of safety plan use (*r* = 0.35, *p* < 0.001). A little over half (58.9%) of clinicians reported working in a setting that has a standard protocol in place for safety plan use. Working in a setting with a standard safety plan protocol was positively associated with overall frequency of safety plan use, *t*(110) = 2.16, *p*=.03. In addition, working in a setting with a standard safety plan protocol was negatively related to completing a safety plan with all patients (*r* = −0.246, *p* < 0.01) and negatively related to completing a safety plan when patients have thoughts about wanting to die but not thoughts of dying by suicide (*r* = −0.269, *p* < 0.01). 

In order to examine whether prior experience with or exposure to suicide was associated with the decision to use safety planning, clinicians were asked to report on multiple types of exposure to suicide. A single item asked if clinicians had previously had a patient die by suicide one time (*N* = 10; 10.8%) and more than one time (*N* = 16, 17.2%). This item was dichotomized into never having a patient die by suicide (*N* = 67; 56.3%) and having a patient die by suicide at least once (*N* = 26; 21.8%). Having a patient die by suicide was not related to completing safety plans with patients in any situation.

The six personal exposure to suicide questions (e.g., “I have an acquaintance who attempted or died by suicide,” “I have attempted or seriously considered suicide”) were aggregated into a single, continuous variable where higher scores indicated more exposure to suicide. Clinicians reported personal exposure to suicide (M = 1.54, SD = 1.60). We dichotomized this variable into those who denied personal experience with suicide (*N* = 45, 37.8%) and those who reported having personal experience with suicide (*N* = 52, 43.7%). Personal exposure to suicide was related to completing a safety plan when a patient (1) has identified a method for suicide and has thoughts of dying by suicide X^2^ (1) = 8.95, *p* < 0.01, with a medium effect size (Cramer’s V = 0.35, *p* < 0.01), (2) has developed a suicide plan X^2^ (1) = 4.93, *p* < 0.05, with a small effect size (Craver’s V = 0.26, *p* < 0.05), and (3) has begun preparing for suicide X^2^ (1) = 6.85, *p* < 0.01, with a medium effect size (Cramer’s V = 0.31, *p* < 0.01). Personal exposure to suicide was not related to completing a safety plan when a patient (1) has thoughts about wanting to die, but not thoughts of dying by suicide X^2^ (1) = 0.91, *p* = 0.34, (2) has thoughts about ending their lives by suicide X^2^ (1) = 2.21, *p* = 0.12, (3) has previously made a suicide attempt, but has not had recent thoughts of suicide X^2^ (1) = 0.01, *p* = 0.90. Personal exposure to suicide was not related to completing a safety plan with all patients X^2^ (1) = 1.42, *p* = 0.23.

## 4. Discussion

The current project investigated mental health providers’ perceptions, training, and experiences with suicide safety planning with at risk patients. Suicide safety planning has become an important aspect of evidence-based, suicide-specific interventions that can be implemented in a variety of service delivery systems [11]. As safety planning may continue to proliferate as an integral clinical tool in preventing suicide, a better understanding of provider experiences and potential obstacles may assist in the intervention’s successful implementation. 

Results of this study demonstrated that the vast majority of the providers surveyed had training in suicide safety planning. Not surprisingly, most providers also indicated feeling at least somewhat comfortable creating suicide safety plans. Core elements of safety planning, such as identifying warning signs and means safety strategies, are routinely used. These high rates of training and comfort with safety planning are promising, but it should be noted that almost 70% of the sample indicated a need for further training. Although we are unable to determine the reason for this discrepancy, it could be that providers are comfortable with completing some or most aspects of the safety plan (e.g., internal coping skills, emergency resources) but are less comfortable completing others (e.g., means safety) and thus desire more training in this specific aspect of safety planning.

One area in which further training could be provided is through the proliferation of free training manuals and safety planning-based applications. Less than 35% of providers were aware of the free Stanley and Brown [10] suicide prevention training manual. Dissemination of the manual, as well as free online courses, such as the Counseling Access to Lethal Means course [6], may help augment formal training in safety planning and promote increased efficacy for providing the intervention. 

Ideally increased training opportunities would result in the more rigorous practice of safety planning. Although the core elements of the manualized Safety Planning Intervention were used frequently, common clinical practices, such as encouraging patients to share safety plans with family/friends or practicing safety planning with a patient, were used less often. Certainly, other factors likely play a role in why these practices are not commonly used (e.g., time constraints). For example, within the current study, clinicians reported spending approximately 25 min on safety planning. It could be that certain aspects of safety planning are omitted or rushed due to the limited amount of time many providers have with patients, especially in hospitals where are primarily charged with patient triage. It may be that further training with safety planning may increase the use of these practices. Although such practices align with the extant literature on the role of skills practice in cognitive-behavioral therapy [21], the available empirical data do not address the role of additional practice elements in the efficacy of safety planning, specifically.

Another factor that appeared to play a role in provider experience with safety planning was exposure to suicide. Although providers who had had patients die by suicide did not differ in safety plan decision-making, providers who indicated one or more personal exposures to suicide (e.g., losing a friend to suicide, personally experiencing suicidal thoughts or attempts) were more likely to complete safety plans with patients. This result corresponds to studies that indicate that clinical decision-making regarding the treatment of those struggling with suicide-related concerns can be impacted by a myriad of personal factors of the provider [17,18]. Variation in when safety planning is implemented may indicate a need for clearer recommendations regarding which clinical presentations should prompt safety planning and for greater discussion of how attitudes towards suicide may impact patient care. Taken together, we suggest that training in and implementation of safety planning interventions take into account factors which may affect the fidelity of the intervention, such as personal experiences with suicide, experience with the intervention, and environmental obstacles (e.g., time limitation). Training in safety planning may benefit from a focus on the manualized safety plan intervention [10], as only about 1/3 of participants were familiar with this manual. Additionally, training in safety planning might benefit from explicitly addressing potential biases in provider decisions to use safety planning, such as previous provider experiences. 

### Limitations and Future Directions

Interpretation of study results should be made while considering the specific sample and methodological limitations. Regarding the study sample, the vast majority of participants endorsed having an advanced degree in social work, counseling, or psychology. Although providers from these disciplines are likely to interact with at-risk patients, they may also be more likely to have had training in safety planning compared to those from other professions that interact with at-risk patients (e.g., primary care or emergency physicians). It is possible then that high rates of comfort and training in the provision of safety planning are, in part, due to the disciplines represented by study participants. Similarly, approximately one-fifth of participants indicated being students, which could have impacted our results. For example, students may have less exposure working with patients believed to be at risk for suicide, and thus feel less comfortable completing suicide specific interventions such as safety planning.

Regarding study methodology, the use of mental health and suicide prevention-related listservs to recruit providers likely created a restriction in range of many important variables. For example, it may be that more providers with advanced degrees and years of clinical experience frequent listservs such as these compared to providers with less years of training or interest in mental health treatment. Thus, these results may represent the experiences of those most highly trained and familiar with safety planning. Regarding training in safety planning, the survey inquired as to whether participants had received training in safety planning but did not specify formal training (e.g., roleplay training) versus more informal training (e.g., training from a supervisor). It could be that the kind of training that participants received in safety planning had an effect on subsequent comfort and decision-making, which is a question that should be researched in the future. Additionally, objective measures of provider use of suicide safety plans with patients would be a valuable indicator of provider knowledge, skill, and fidelity to the intervention. As a result, it is unknown whether participants used the Stanley and Brown (2012) safety planning template [10], which could have important implications (e.g., handwriting a safety plan from memory could lead to accidental omission of key components)”. Finally, the measures used in this study were developed specifically to answer study questions; therefore, psychometric data was not available. 

Future research should also consider a wider array of factors that may be associated with safety plan use. For example, research may want to consider clinician biases surrounding the need for safety planning (e.g., are clinicians more likely to implement safety planning with men? Racial majority individuals? Teenagers vs adults? Does fear of liability or previous exposure to litigation affect clinician decision-making?). Similarly, future research should evaluate decision-making processes around the circumstances in which providers use safety planning. Research may also wish to consider outcomes other than safety plan use, such as safety plan quality and completeness [15]. For example, factors such as regular updating of the safety plan and accessible storage of the safety plan are important aspects of successful safety plan implementation and could be investigated in future work. Future work would also benefit from a more detailed description of the setting in which clinicians worked, as this may affect clinician experiences with safety planning. For example, a clinician who works at a Veterans Affairs hospital, where safety planning usage is required, may have different experiences than someone who works at a hospital with no specific procedure regarding safety planning.

Finally, future research should examine the clinical decision-making process and how best to link SRA tools (such as the C-SSRS, SIQ-JR, and BSS) for recommendation for when to implement safety planning.

## 5. Conclusions

Data supports the use of suicide safety planning to increase the safety of at-risk patients [11]. The results of the current study support that many providers routinely complete suicide safety planning and feel relatively comfortable in providing this part of suicide-specific care. However, these data also identify gaps in training and inconsistency in the use of safety planning, indicating the need for additional training opportunities and for clear implementation guidelines. In particular, future efforts might focus on elements of good clinical practice in safety planning (e.g., when to involve others, practicing safety plan use), determining when to implement safety planning based on patient risk level and how safety planning can be best practiced in session and/or with supportive others. These data provide important considerations for those developing training protocols in suicide safety planning as well as those providers being trained in the intervention. Rigorous training and successful implementation of suicide safety planning may help healthcare facilities reduce the burden of suicide in their communities [22].

## Figures and Tables

**Table 1:** Clinicians’ estimated percent of the time they include major components of the safety plan.

Item	*M*	*SD*	Range
When creating a safety plan, how often do you…			
1. Include individual warning signs/triggers of a crisis	84.00	26.64	0–100
2. Include individual coping skills	94.58	12.36	21–100
3. Include social coping skills/social distraction	90.32	16.32	0–100
4. Include social help-seeking	89.86	17.76	0–100
5. Include emergency resources/emergency contacts (e.g., hotlines, on-call clinicians, etc.)	97.34	8.65	41–100
6. Include means restriction (e.g., limiting access to firearms, pills, etc.)	86.26	24.35	0–100
7. Identify and write down reasons for living	74.16	31.39	0–100
8. Have the patient write down their safety plan themselves	61.05	33.87	0–100
9. Create physical reminders (e.g., a copy of the safety plan to keep with you)	84.55	26.73	0–100
10. Create a Hope Box	12.23	23.86	0–100
11. Create a Virtual Hope Box or use another safety planning app	12.72	25.09	0–100
12. Use of a safety plan template	69.64	39.32	0–100
13. Provide instruction on the proper use of the safety plan	75.41	36.12	0–100
14. Practice using the safety plan in session	46.07	39.75	0–100
15. Share the safety plan with a parent/guardian, significant other, or family member	55.64	36.99	0–100

**Table 2:** Frequency of clinician safety plan completion.

Clinician Completes Safety Plan…	Frequency	Percentage
with all patients	13	11.5
when patients have thoughts about wanting to die, but not thoughts of dying by suicide	57	50.4
when patients have thoughts about ending their lives by suicide	98	86.7
when a patient has identified a method for suicide and has thoughts of dying by suicide	83	73.5
when a patient has begun preparing for suicide	76	67.3
when a patient has developed a suicide plan	81	71.7
when a patient has previously made a suicide attempt, but has not had recent thoughts of suicide	47	41.6
when a patient has previously made a suicide attempt and is having thoughts of suicide	88	77.9

Note: *N* = 119.

## Supplementary Materials

The following are available online at https://www.mdpi.com/1660-4601/17/18/6444/s1. File S1: Supplementary Survey Questions.
