# Supplementary material for: Suicide Safety Planning: Clinician Training, Comfort, and Safety Plan Utilization
Source: Int J Environ Res Public Health. 2020 Sep 4;17(18):6444. doi: 10.3390/ijerph17186444 (PMC7559434; doi:10.3390/ijerph17186444)
Supplement: Supplementary file 1 [file ijerph-17-06444-s001.pdf]

Safety Planning Questionnaire

Thank you for your interest in our survey!

First, we would like to ask several questions ask about your experience and training in safety planning for suicide prevention.

We use "safety planning" to refer to any efforts to establish, with clients, a procedure, protocol, or plan for handling suicidal thoughts or urges. Safety plans seek to assist clients in "getting through," or navigating a suicidal crisis or period of suicidal thoughts safely, without acting on those thoughts. Safety plans frequently use self-distraction, coping skills, help-seeking behavior, means restriction, or some combination of those strategies. Safety planning may be done using an established template, on paper or electronically, or may be conducted informally with clients and/or families.

Do you think completing a safety plan with a client has the potential to save his or her life?

- Definitely yes
- Probably yes
- It might or might not
- Probably not
- Definitely not

Have you received training in safety planning?

- Yes
- No

If yes, did you receive training in safety planning as part of your education?

- Yes
- No
- Not applicable - I have not received training in safety planning.

Have you ever read or are you familiar with the Stanley & Brown manual for safety plans? (Available here: www.mentalhealth.va.gov/docs/va_safety_planning_manual.pdf)

- Yes
- No

How frequently would you say you use safety planning for suicide prevention with your clients?

- Never
- Rarely
- Sometimes
- Frequently
- Always/Nearly Always

How comfortable would you say you are with safety planning for suicide prevention?

- Extremely comfortable
- Somewhat comfortable
- Somewhat uncomfortable
- Extremely uncomfortable

How comfortable are you in completing the following sections of standard safety plans?

	Very uncomfortable	Somewhat uncomfortable	Somewhat comfortable	Very comfortable
Identifying warning signs	•	•	•	•
Identifying coping strategies and distracting activities	•	•	•	•
Identifying social supports	•	•	•	•
Identifying means safety strategies and helping make a client's environment safe	•	•	•	•

Do you feel the need for additional training in safety planning for suicide prevention?

- No, definitely not
- No, not really
- Yes, a little
- Yes, definitely

On average, how long does it take you to complete a safety plan? (in minutes). ________

When creating a safety plan, how often do you...

	Never (0%) … Always (100%)<br>0 10 20 30 40 50 60 70 80 90 100
Include individual warning signs/triggers of a crisis	
Include individual coping skills	
Include social coping skills/social distraction	
Include social help-seeking	
Include emergency resources/emergency contacts (e.g., hotlines, on-call clinicians, etc.)	
Include means restriction (e.g., limiting access to firearms, pills, etc.)	

Identify and write down reasons for living	
Have the client write down their safety plan themselves	
Create physical reminders (e.g., a copy of the safety plan to keep with you)	
Create a Hope Box?	
Create a Virtual Hope Box or use another safety planning app?	
Use of a safety plan template	
Provide instruction on the proper use of the safety plan	
Practice using the safety plan in session	
Share the safety plan with a parent/guardian, significant other, or family member	

We are also interested in learning about the circumstances that prompt clinicians to create a safety plan with a client. In which of the following situations would you be likely to create a safety plan with a client? (please select all that apply)

- ☐ I complete a safety plan with all of my clients
- ☐ When clients have thoughts about wanting to die, but not thoughts of dying by suicide
- ☐ When clients have thoughts about ending their lives by suicide
- ☐ When a client has identified a method for suicide and has thoughts of dying by suicide
- ☐ When a client has developed a suicide plan
- ☐ When a client has begun preparing for suicide (e.g., gathering needed items, writing a will, writing a suicide note)
- ☐ When a client has previously made a suicide attempt, but has NOT had recent thoughts of suicide
- ☐ When a client has previously made a suicide attempt and is having thoughts of suicide

Is there a standard plan in place in your work setting for when a suicide safety plan should be created? (e.g., at a certain risk level or in response to certain types of suicidal thoughts)

- Yes
- No

If none of the above questions captured when you decide to create a safety plan, please tell us more about when you decide to create a safety plan with a client.

Please provide your age. _____

How do you identify your gender?
- Male
- Female
- Non-Binary/Third Gender
- Prefer to self-describe: ____
- Prefer not to say

How would you identify your race/ethnicity? (Please select all that apply.)
- Caucasian
- African American/Black
- Asian American
- Native American / Alaskan Native
- Native Hawaiian / Pacific Islander
- Hispanic
- Prefer not to answer
- Other (please specify) ____

Please selected your highest completed degree.
- Associate's Degree
- Bachelor's Degree
- Master's Degree
- Doctoral Degree
- Other (please specify) ____

Please select your occupation:
- Social Worker
- Therapist/Counselor/Psychological Associate
- School/Guidance Counselor
- Psychologist
- Psychiatrist
- Crisis Center Worker
- Student (please provide discipline of study) ____
- Other (please specify) ____

How would you describe your primary setting?
- Crisis Center
- Outpatient/Community Clinic
- Inpatient/Psychiatric Hospital
- School Counseling Department
- Academic Medical Center
- School
- Other (please specify) ____

How many years of experience do you have in providing mental health services? ____

Have you ever lost a client to suicide?

- Never
- Once
- More than once

Please read each of the following statements carefully and select all of the statements that apply to you.

- ☐ I have never been exposed to anything related to suicide.
- ☐ I have observed suicide in a movie or television show.
- ☐ I have seen a documentary on suicide.
- ☐ I know a colleague who attempted or died by suicide.
- ☐ I have provided services to someone who attempted or died by suicide.
- ☐ I have an acquaintance who attempted or died by suicide.
- ☐ I have a relative who attempted or died by suicide.
- ☐ I have a close friend who attempted or died by suicide.
- ☐ I have lived with someone who attempted or died by suicide.
- ☐ I have attempted or seriously considered suicide.
